# Implementing the Co-Immune Open Innovation Program to Address Vaccination Hesitancy and Access to Vaccines: Retrospective Study

**DOI:** 10.2196/32125

**Published:** 2022-01-21

**Authors:** Camille Masselot, Bastian Greshake Tzovaras, Chris L B Graham, Gary Finnegan, Rathin Jeyaram, Isabelle Vitali, Thomas Landrain, Marc Santolini

**Affiliations:** 1 Just One Giant Lab Association Paris France; 2 Center for Research and Interdisciplinarity INSERM U1284 Universite de Paris Paris France; 3 IXL Editorial Kildare Ireland; 4 Sanofi Gentilly France

**Keywords:** open science, open innovation, programmatic research, collective intelligence, web based, immunization, vaccination access, vaccine hesitancy, innovation, vaccine, public health, access, framework, participatory, design, implementation

## Abstract

**Background:**

The rise of major complex public health problems, such as vaccination hesitancy and access to vaccination, requires innovative, open, and transdisciplinary approaches. Yet, institutional silos and lack of participation on the part of nonacademic citizens in the design of solutions hamper efforts to meet these challenges. Against this background, new solutions have been explored, with participatory research, citizen science, hackathons, and challenge-based approaches being applied in the context of public health.

**Objective:**

Our aim was to develop a program for creating citizen science and open innovation projects that address the contemporary challenges of vaccination in France and around the globe.

**Methods:**

We designed and implemented Co-Immune, a program created to tackle the question of vaccination hesitancy and access to vaccination through an online and offline challenge-based open innovation approach. The program was run on the open science platform Just One Giant Lab.

**Results:**

Over a 6-month period, the Co-Immune program gathered 234 participants of diverse backgrounds and 13 partners from the public and private sectors. The program comprised 10 events to facilitate the creation of 20 new projects, as well as the continuation of two existing projects, to address the issues of vaccination hesitancy and access, ranging from app development and data mining to analysis and game design. In an open framework, the projects made their data, code, and solutions publicly available.

**Conclusions:**

Co-Immune highlights how open innovation approaches and online platforms can help to gather and coordinate noninstitutional communities in a rapid, distributed, and global way toward solving public health issues. Such initiatives can lead to the production and transfer of knowledge, creating novel solutions in the public health sector. The example of Co-Immune contributes to paving the way for organizations and individuals to collaboratively tackle future global challenges.

## Introduction

### Background

As the world faces a rise in the number of complex challenges that threaten the resilience of our economic, environmental, social, and health systems, we observe a shift toward more collaboration and openness in the way science and innovation is performed [1-3], bringing governments, civil society, and the private sector closer. Examples of this include the efforts made to accelerate society’s progress toward the Sustainable Development Goals (SDGs) [4] and the fight against pandemics, such as COVID-19 [5]. Yet, access to vaccines and vaccination hesitancy remain as some of the complex challenges to be addressed in order to achieve universal health coverage [6].

Immunization is one of the most cost-effective interventions to protect oneself and others from infectious diseases [7] and saves between 2 million and 3 million lives per year [8].

Yet, the annual death toll for vaccine-preventable diseases stands at 1.5 million, and large gaps in coverage persist, not only between countries but also within their territories [7]. In particular, the World Health Organization (WHO) listed vaccine hesitancy among the top 10 global health threats for 2019 [9]. Continuing global efforts to leave no one behind may be a long-standing challenge [10] when new information technologies and social media platforms are both part of the problem [11] and the solution. More recently, the COVID-19 pandemic demonstrated the repertoire of logistical and administrative challenges to the deployment and administration of vaccines, especially in low-resource settings [12].

In response, the WHO Global Vaccine Action Plan 2011-2020 [7] committed 140 countries and 290 organizations to promoting and prioritizing greater collaboration between governments, nongovernmental organizations, the private sector, and all citizens to address outbreaks of vaccine-preventable diseases. Additionally, a number of new digital and open innovation initiatives have been launched: the WHO has developed the Vaccine Safety Net [13], a network of websites about vaccination; health authorities in Canada have developed a school-based quiz to educate children about immunology and vaccines [14]; Finland is testing a computer game to communicate the benefits of human papillomavirus vaccination [15]; a project in India uses digital necklaces to record children’s immunization history [16]; and the global Vaccination Acceptance Research Network has been established [16].

Global health guidelines showcase the positive outcomes of social participation for universal health coverage [17], which include more meaningful dialogue, more sustainable solutions, and more trust from citizens in health system institutions or in the decisions that are made. Indeed, there is room for more initiatives that allow people to genuinely co-design solutions in a multidisciplinary manner during and following pandemics [18]. Hence, the number and sustainability of these types of initiatives could be amplified by fostering increased collaboration with nonacademic citizens in the creation and development of solutions in an open innovation framework [19]. This is the gap that Just One Giant Lab (JOGL) is proposing to fill with the Co-Immune program.

Citizen science is an emerging and highly diverse practice that can be broadly defined as the general public being involved in the process of doing research [20]. Research has demonstrated that intensity and diversity of collaboration positively affect the quality [21] and productivity [22] of research, while positively impacting the knowledge integration from participants [23]. Likewise, participant transdisciplinarity [24] seems critical to generating innovative outcomes [25] and dealing with complex real-world problems [26]. Such mechanisms are often at play in the field of citizen science, promising to transform the knowledge generation landscape by tapping into networks of nonacademic citizens [26,27] in a new social contract for this kind of research [28]. Citizen science has the potential to expand the number of individuals contributing knowledge and ideas, transform how hypotheses are generated, and transform how data sets are analyzed. Such approaches have already been applied to investigate individual diseases through patient-led research [29,30] and public health challenges, such as the epidemiology of cancer [31-33].

Other approaches to create and develop knowledge and solutions to complex challenges are slowly entering the mainstream. In particular, hackathons, challenge-based approaches, and the participation of citizens in science have been flourishing over the last two decades [34], especially within the natural sciences [35] and, more recently, within medical sciences, public health, and population-health research [36,37].

Hackathons are short, intensive, and collaborative events that are designed to prototype solutions addressing a specific problem. They originated in the early 2000s in digital and tech fields and have been adapted to address more complex challenges in global health [38-40]. Such initiatives are not without pitfalls: they suffer, by design, from the lack of paths to sustainability for the projects they launch [41]. In response to such criticisms, there are increasing efforts, such as the “Make the Breast Pump not Suck” hackathon and “Trans*H4CK,” to improve hackathon methodology by working directly with affected communities [41]. Several initiatives, such as a Massachusetts Institute of Technology collaborative design studio, provide insights into hackathon methods [42] to facilitate better hackathons [43,44]. More recently, multiple entities have engaged in organizing hackathons to address the COVID-19 crisis [45,46].

Challenge-based approaches, which provide frameworks for learning while solving real-world issues, have also been on the rise in global health and have proven to be efficient for generating innovative solutions and for incentivizing mass community engagement [45]. For example, the potential of participative models to address complex questions, along with the power of contests to offer a structure that catalyzes this work, has been exhibited by the Epidemium initiative on cancer epidemiology [46].

Despite the numerous tools and technologies created to facilitate collaboration in citizen science projects, challenges remain. These include the issues of the complementarity, coherence, and diffusion of these initiatives [34] to efficiently address international policies and local needs, as the local adoption of hackathon solutions often remains low [47].

Therefore, the promotion of transdisciplinarity and citizen science in an open innovation framework, coupled with methods such as hackathons, and a challenge-based approach represent an opportunity to address current complex challenges of vaccination that would overcome the limits of either solution alone. In this paper, we describe the design, implementation, and outputs of Co-Immune, a collaborative open innovation program that was run in 2019 to address vaccination hesitancy and access to vaccination.

### Objectives

Co-Immune’s aim was to develop an environment that favors the creation and development of citizen science and open innovation projects addressing the contemporary challenges of vaccination in France and around the globe. This program had four specific objectives: (1) to foster a collaborative, open, and transdisciplinary dynamic; (2) to promote the emergence of accessible knowledge and innovative solutions; (3) to support participants in the elaboration and development of their project; and (4) to disseminate the outputs and results in an open science framework. In this study, we describe the methodology of Co-Immune and its implementation, and we present its key outcomes.

## Methods

### Design

The overall program duration was 10 months (March 2019 to January 2020), divided into 6 months of preparation and 4 months of rollout of activities that included offline and online events, support for the development of citizen science projects, and assessment and awards for projects participating in the challenge-based competition. The main outputs of the program were projects, categorized as leading to (1) knowledge production, if they performed data analysis or generated new knowledge, whether it was context specific, generic [48], or knowledge transfer [49]; or (2) solutions, such as hardware, software, and interventions.

Co-Immune was coordinated online through the JOGL platform [50] and supported by 13 partners from the public and private sector (Table S1 in Multimedia Appendix 1). The challenge-based nature of the program was designed to be an incentive for teams and participants to continue developing their projects after hackathon events or to create their project on JOGL at any other time.

The governance of Co-Immune was designed to provide freedom for projects to develop innovative solutions while ensuring their compliance with local and international regulations and consideration of ethical and scientific integrity. To this end, we constituted the independent Committee for Ethics, Science and Impact (CESI), which issued an opinion on the rules of participation in the program and validated the strategic orientation of the program. Public health priorities were identified based on a literature review and divided between two main challenges to streamline participants’ work: vaccination coverage and vaccination hesitancy. They were then validated by the CESI. In addition, through a series of semistructured interviews, experts at the 7th Fondation Merieux Vaccine Acceptance conference [51] identified eight specific issues to address and potential room for solutions. The CESI also participated in the co-elaboration of the assessment grid, which was used as a base to grant nonmonetary prizes to projects in December 2019.

To be eligible for a prize, a project was required to have created a comprehensive description of their initiative on the JOGL platform and a video pitch. This material was provided to experts in charge of the assessment.

### Participant Recruitment

Participants were recruited through our network of partners from around the globe and social media communication. Participation was open to everyone above the age of 18 years, if they had agreed to follow the participation rules validated by the CESI. Participants could take the role of “project leader” or “contributor.”

### JOGL Platform

**Figure 1:**
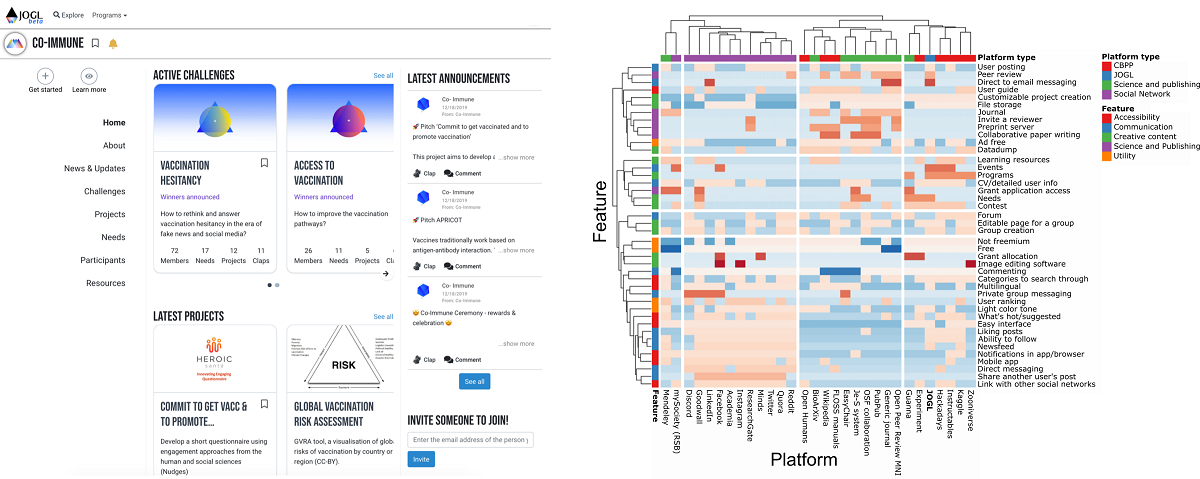
Overview of the Just One Giant Lab (JOGL) platform. The image on the left is a screenshot of the JOGL platform. The right-hand image is a heatmap of feature presence across popular online tools. For each platform (columns), we numerically encoded the presence (1) or absence (0) of each feature (rows). Then, for each element, we computed a Z score by standardizing values across platforms, represented here by the color spectrum: blue (low) to red (high). CBPP: citizen-based peer production network (ie, citizen science platform); CV: curriculum vitae; Je-S: Joint Electronic Submissions; MNI: Montreal Neurological Institute; OSF: Open Science Framework; RSB: Royal Society of Biology.

Co-Immune participants used the JOGL platform to document their projects and recruit collaborators throughout the course of the program. JOGL is a decentralized mobilization platform designed for use in collaborative research and innovation (Figure 1). Within the JOGL platform, users can create a profile and declare their skills. Once registered, they can create or join projects, follow the activity of other members, post on their project feed, and comment on other posts. They can also highlight needs for a project they are part of, specifying skills that can help to solve project problems. We compared the JOGL features to those of other online platforms for citizen science, social networking, and science and publishing through a cluster analysis (see Figure 1 as well as the supplementary method and Figure S1 in Multimedia Appendix 1), indicating that the platform is functionally similar to other platforms in the space and is suitable to hold a citizen science program such as Co-Immune.

### Implementation

**Figure 2:**
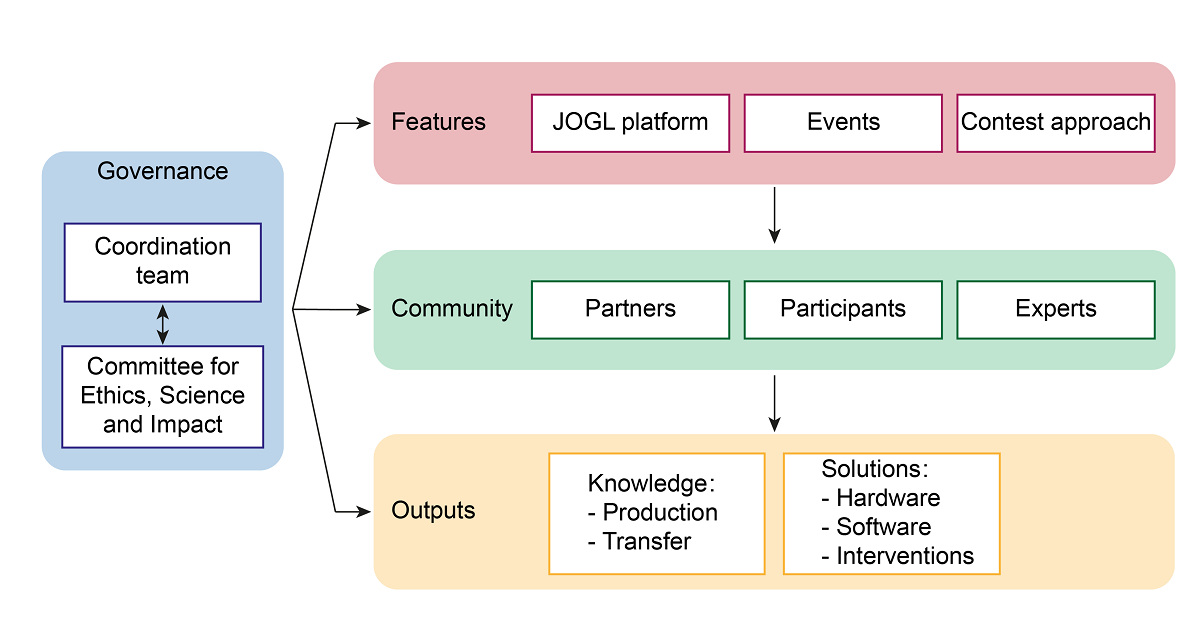
Workflow of the Co-Immune program design. JOGL: Just One Giant Lab.

The Co-Immune program was realized through an interrelated and interacting set of technological and social features (Figure 2). Our coordination team implemented the larger program (ie, events, online platform, and contest approach) and helped to recruit a community of partners and participants who interacted with each other and were supported in their efforts through the high-level design features. With support from the governance structure of the Co-Immune program, the individual projects managed to provide outputs that included knowledge production and transfer as well as solutions, such as hardware, software, and interventions.

### Building an Open Community

To build the community, we contacted organizations involved in a wide range of domains before the launch of the program, thereby creating a first pool of contributing professionals and students. We also recruited participants via the organization of events, typically in the evening, aimed at creating projects, fostering collaboration among participants to address project needs, and providing mentorship. To facilitate the coordination of the community, all participants were required to use the JOGL platform to describe their projects, form teams, list their needs, and initiate collaboration.

In order to create a supportive and collaborative environment for the participants, we reached out to various organizations to establish partnerships. Our intention was two-fold: (1) to facilitate the participation of the organizations’ students and employees as participants or mentors by involving their institution and (2) to enhance the sustainability of projects after the course of the program by connecting them with potential partners at the early stage of their development.

**Figure 3:**
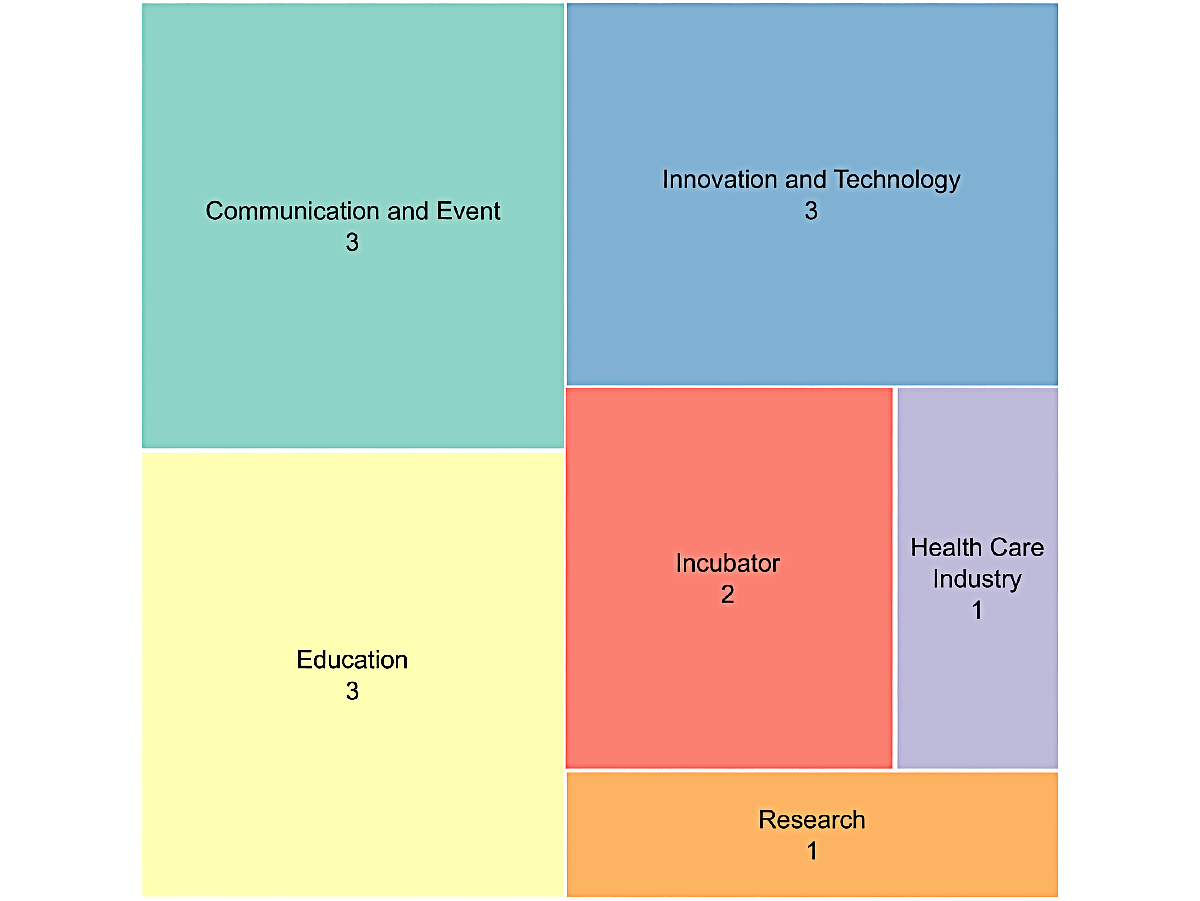
Treemap representing the domains of action of the 13 Co-Immune partners.

The 13 partners operated in the health, technology, and social sectors, and included research, innovation, and education organizations, as well as professional networks, incubators, and communication specialists (Figure 3). The number of partners grew over the life span of the initiative and were often suggested by existing partners or through connections made during events.

We organized 10 offline and online events between October and December 2019 (Table 1). Participants for events were recruited through social media and mailing lists leveraging our network of partners. Among the four on-site events that were organized, two were hackathons aimed at motivating participants to join the program, while the other two were aimed at fostering collaboration around the most advanced projects. Their median duration was 2.25 (IQR 2) hours.

The facilitation of the hackathon-style events relied on the use of participatory and collective intelligence design and problem-solving techniques [52]. In particular, participants were encouraged to form multidisciplinary teams including both professionals and students.

Three partners in Paris—Epitech, the Wild Code School, and the Center for Research and Interdisciplinarity (CRI)—co-organized and hosted events for their students, respectively, in their engineering, coding, and life science and education schools. Other partners—Kap Code, Excelya, and CorrelAid—mobilized their teams to act as mentors during these events. A total of 14 mentors attended events, and five came to more than one event.

In addition, we organized four 1-hour online events. The first was an opportunity to share information about Co-Immune with people around the globe. Another event discussed best practices to document open science projects. Finally, two events focused on the resolution of needs of single projects (Table 1 [53,54]).

**Table 1:** Co-Immune events.

Name	Mode; type; location	Duration (hours), n	Objective	Design; supporting partners (if applicable)	Participants, n
Launch	Offline; ceremony; CRI^a^, Paris	3	Gather the initial community	Presentation of the program design, features, timeline, and partners, as well as networking	60
OpenJOGL^b^; Co-Immune	Online	1	Q&A^c^ session on the program	Presentation of Co-Immune and questions and answers	3
Sprint; open data	Offline; hackathon; CRI, Paris	2.5	Build community, create projects, and create data repositories	Statement of the problem (videos of experts), team formation and effort, mentoring, and publication of results on the JOGL platform; supported by CRI and CorrelAid	25
OpenJOGL; Vaccination Awareness Escape Game [54]	Online	1	Foster collaboration around single projects	Pitch of the project and its needs, feedback from experts, and questions and answers	7
Sprint; project creation	Offline; hackathon; CRI, Paris	4	Build community and create multidisciplinary projects	Statement of the problem (videos of experts), ice breaker, multidisciplinary team formation and effort, mentoring, presentation of results, vote for the most promising projects, publication of results on the JOGL platform, and networking; supported by CRI, Epitech, Wild Code School, CorrelAid, and Excelya	22
Sprint; open data	Offline; hackathon; Wild Code School, Paris	3	Accelerate the development of projects related to data science	Selection of a project by participants among the two choices available, team formation and effort, mentoring, presentation of results, publication on the JOGL platform, and networking; supported by Wild Code School, CorrelAid, and Excelya	15
Sprint; open data	Offline; hackathon; Epitech, Paris	3	Build the community, create projects, and accelerate the development of one project using Twitter data	Statement of the problem, selection of a project by participants among the four choices available (including one already existing project), team formation and effort, mentoring, presentation of results, vote for the most promising project, publication of results on the JOGL platform, and networking; supported by Epitech, Kap Code, Excelya, and CorrelAid	35
OpenJOGL; HERA^d^: A Health Platform for Refugees [53]	Online	1	Foster collaboration around single projects	Pitch of the project and its needs, feedback from experts, and questions and answers	7
OpenJOGL; better documentation for better collaboration	Online	1	Help teams document their projects in the most open and reproducible way	Expert presentation on best practices for documenting open science projects, presentation of Co-Immune expectations for documentation, and questions and answers	13
Closing ceremony	Offline; ceremony; CRI, Paris	2	Close the Co-Immune program	Presentation of the main outputs of the program and awards for the best projects	70

^a^CRI: Center for Research and Interdisciplinarity.

^b^JOGL: Just One Giant Lab.

^c^Q&A: question and answer.

^d^HERA: Health Recording App.

### Co-Immune Experts: CESI Members, Mentors, and Interviewees

Individuals who were considered “experts” included all the CESI members as well as experienced professionals of a certain field who attended events and provided technical guidance to teams as “mentors.”

The CESI members were sought to represent the diversity of stakeholders involved in advancing access to vaccines and reducing vaccine hesitancy. By choosing interviewees who were researchers specializing in the challenges of access to vaccines and vaccination hesitancy, we aimed at benefiting from their expert understanding of the issues and of the priorities to be addressed to streamline the work of participants around particular problems. Finally, we grew the pool of mentors over the span of the program to best match their expertise with the needs of the projects in an agile manner.

**Figure 4:**
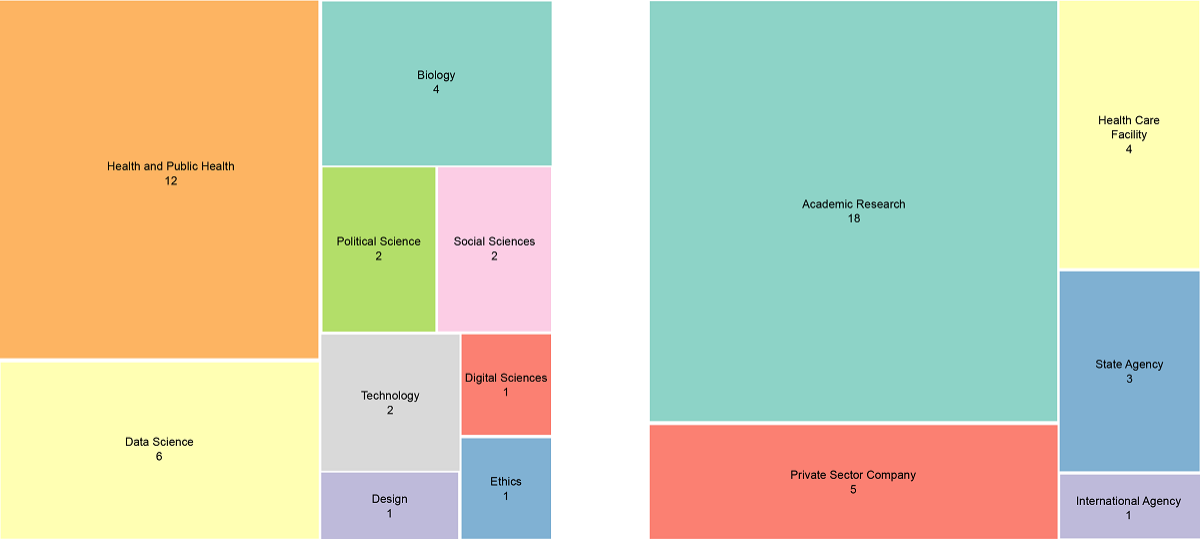
Treemap of the 31 Co-Immune experts: domains of expertise (left) and affiliations (right).

Overall, the mentors’ domains of expertise ranged from biology to social sciences, design, technology, and data science (Figure 4). One-third of them were working as health or public health professionals.

The CESI consisted of eight volunteer members and included virologists, pharmacists, health economists, experts in the digital sciences and ethics fields, and biologists; members were working at international, national, and local levels of the health system. All of them worked for public or nonprofit organizations. Interviewees were mostly researchers in social sciences and medical practitioners.

### Co-Immune Project Assessment

The assessment of projects by experts was designed to be an opportunity for learning and growth. To be assessed, teams were asked to provide a video pitch summarizing their project and detailed documentation on their project page on the JOGL platform, including links to their open access data and code. Project assessment was performed through a grid that was codeveloped by JOGL and the CESI. In addition to grades, teams received detailed feedback on their projects.

The assessment grid was based on a literature review of project evaluation standards and consisted of 10 questions graded from 0 to 5 (Multimedia Appendix 2). Three areas were assessed: the approach, the implementation strategy, and the impact. First, the assessment of the approach included the following: (1) clarity and relevance of the problem and alignment with the program scope, (2) fit between the approach and methodology and the problem statement, and (3) innovation potential (ie, the project introduces groundbreaking objectives, novel concepts, or approaches). Second, the implementation strategy was assessed using following the criteria: (1) state of progress toward set goal (ie, state of advancement), (2) clarity and relevance of the timeline and needs for the future (ie, major tasks and milestones), and (3) project actively engages and aligns with all relevant stakeholders. Finally, the assessment of the impact covered the following: (1) clarity and relevance of the criteria used to measure impact, (2) the extent to which the project considers its ecosystem (ie, ecological, environmental, ethical, and social considerations), (3) sustainability and scalability of the project in the long term, and (4) open and reproducible dissemination strategy. For each of these three categories, JOGL awarded a prize to the project with the best score based on the grades given by reviewers. Additionally, a grand prize was given to the project with the overall highest score. JOGL provided visibility, while two partners also provided an award to a project of their choosing.

### JOGL Platform Data Collection and Analysis

Participants added their professional background, skills, and employment status to the JOGL platform. These data were used to evaluate the composition of the community. All users who joined JOGL during the span of the program were considered to be participants of Co-Immune, as it was the only ongoing program, and all outreach activities were related to it.

To better understand how skills were related across participants, we used a network approach to assess similarity between skills and to get further insights about the global diversity of the community. In this network approach, each declared skill was a node and the skills were considered linked if they co-occurred in a participant. Links were then weighted by the number of participants within which they co-occurred. Gephi 0.9.2 was used to represent the network shown in the skill map of the Co-Immune community, and the modularity algorithm was used with default parameters to compute communities representing the sets of skills that tend to co-occur more together than with other skills. Since these skills are linked through the participants who share them, they can be understood as "participant types" constitutive of the Co-Immune community.

We provide the data related to this study on Zenodo [55]. These data include (1) the link, description, and assessment scores of projects; (2) the profiles of platform users; (3) the description of events; (4) the profiles of experts; and (5) the list and types of partners.

## Results

### Community Growth Through Events

**Figure 5:**
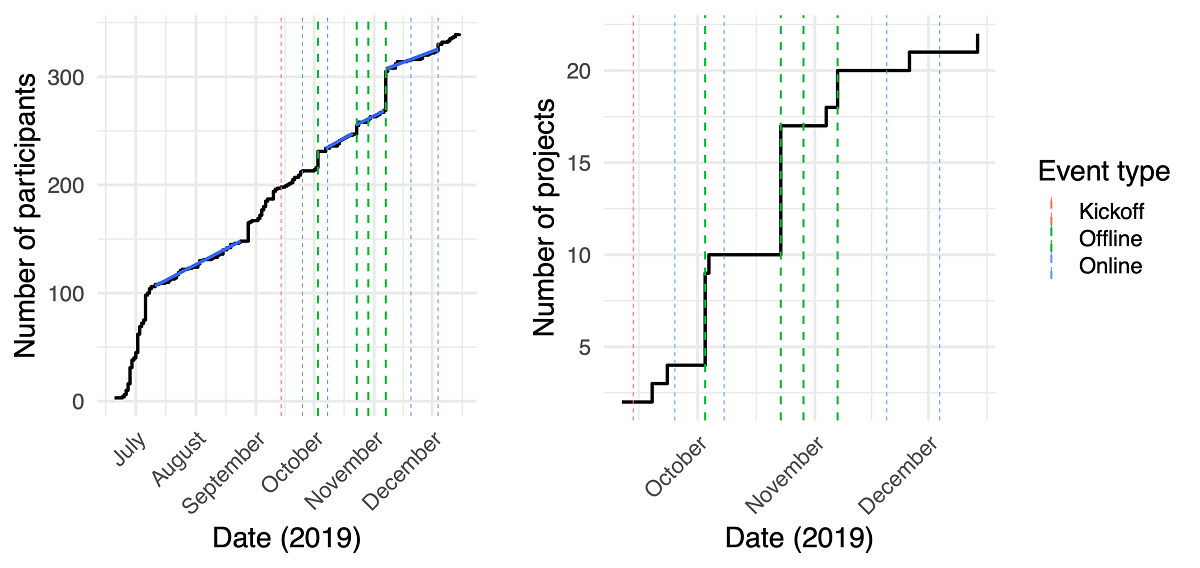
Growth of the number of participants (left) and number of projects (right) over the life span of the program. Dashed bars show when events for community facilitation where held (green: offline events; blue: online events; red: kickoff meeting). Blue lines give a linear fit during the corresponding periods, showing stable growth pre- and postkickoff.

During the program, 234 participants signed up to the platform (Figure 5). The participant growth was mostly linear over the life span of the program (July 10 to December 18, 2019), suggestive of the potential for continued growth if the program had continued. The growth rate outside of events, at around one per day (between 0.86 and 0.98 users/day), was consistent with the prekickoff growth rate (0.94 users/day). This highlights the importance of events (dashed lines in Figure 5) for driving participant enrollment, with the four offline events accounting for 45% of the growth. In total, offline events were responsible for the generation of 82% (18/22) of the projects. The rest consisted of 4 out of 22 (18%) projects created on the platform outside of events and 2 already-existing projects prior to the program.

### Participant Skills and Backgrounds: A Transdisciplinary Community

**Figure 6:**
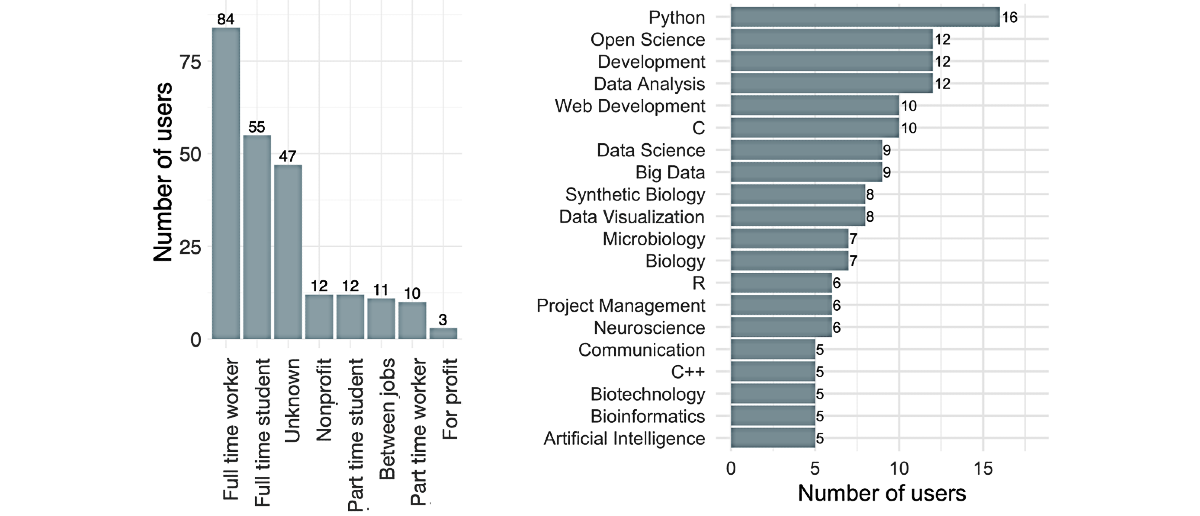
An overview over the Co-Immune community: participant categories (left) and the 20 most represented skills (right) in the Co-Immune community.

Out of the 234 participants, 187 (79.9%) declared their job category. The community was composed of a mix of students (67/187, 35.8%) and workers (94/187, 50.3%), most of whom worked full time (81/94, 86%; Figure 6). Other categories included “between jobs” (n=11), “nonprofit” (n=12), and “for profit” (n=3). Out of the 75 participants who declared their country in their JOGL profile, 57% (n=43) were based in France, with the rest coming from other regions, including the rest of Europe, the Americas, Africa, and Asia.

**Figure 7:**
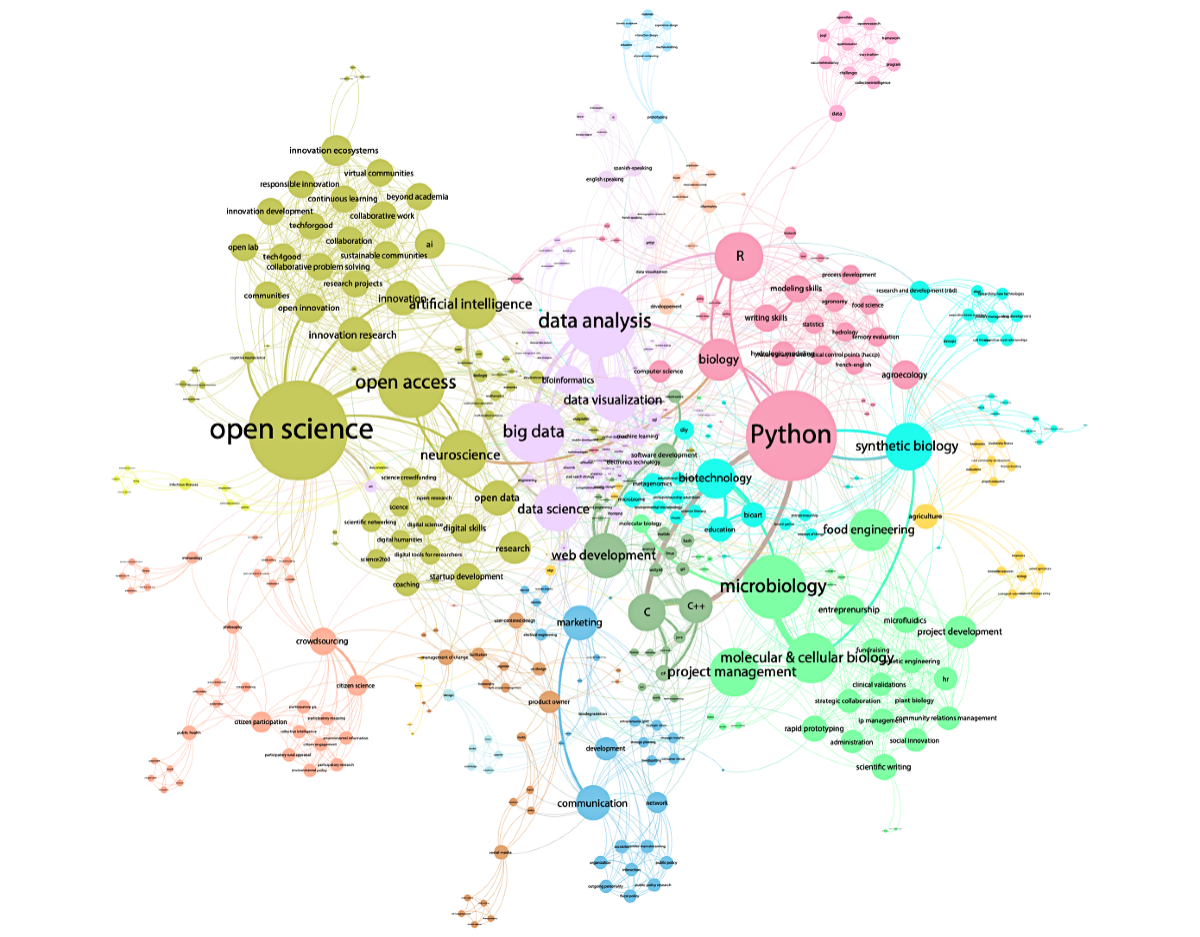
Skill map of the Co-Immune community. Skills are linked if they appear in the profile of the same participant. Link weight indicates the number of participants sharing the skills. Node size indicates weighted degree.

The 234 participants specified a total of 492 unique skills (median 3 [IQR 4.5] skills per participant). We observed a high representation of data science and coding alongside biology, which, altogether, related to the technical skills emphasized during the program (Figure 6). The skill network shows that the community spanned a vast interdisciplinary landscape, from open science to open data and coding, and from project management to biology. The network exhibited the largest connected component of 416 interconnected skills (84.6% of all skills; Figure 7). The modularity maximization (see the Methods section) resulted in the identification of 12 modules corresponding to “participant types” constitutive of the Co-Immune community.

### Co-Immune Project Description

A total of 22 projects were created by 20 project leads, with teams of up to 11 members (Table 2 [52,53,56-75]). Among these, 15 (68%) projects proposed to develop software covering web technologies, mobile apps, algorithms, data lakes, data modeling and analysis, and visualization tools. The other 7 (32%) projects included hardware development and interventions involving biotechnologies, game design, behavioral sciences, education, and communication. Overall, one-third of the projects focused on knowledge transfer.

Among the 15 projects relying on software technology, 11 (73%) aimed at contributing to the production of knowledge by facilitating the analysis of publicly available data; they did this via the use of parsing tools and the creation of repositories (n=3), the analysis of open data (n=3), the development of machine learning tools to extract and analyze Twitter data related to vaccination hesitancy (n=2), and the production of data visualizations (n=3). In particular, more than 40 data sets were identified and collected by 4 projects that were created during the data-centered events. In addition, a database of 2464 tweets, in French, posted over a period of 7 years was made available by a partner, and another data set of 89,979 tweets was gathered by the project Qualitative Analysis of Tweets on Vaccination [56].

Out of the 15 projects above, 4 (27%) used software for knowledge transfer; for instance, the HERA (Health Recording App) project [52] provided educational content and health data storage through its mobile app to improve the monitoring of vaccination and perinatal health among Syrian refugees in Turkey. The Pass It On project [60] focused on role-playing video games directed at health professionals as another method of knowledge transfer. The Neutralizing Information About Vaccines project [70] implemented an algorithm for parsing web pages, helping citizens identify trustworthy content related to vaccines.

A total of 5 projects out of 22 (23%) focused on different interventions (Table 2), including raising awareness about vaccination through an escape game (ie, Vaccination Awareness Escape Game [54]) and communication campaigns on social media (ie, Go Viral! [71]). The HEROIC Santé project [57] developed and tested a short questionnaire using engagement approaches from the social sciences to engage health care professionals and users around the question of flu vaccination. Finally, one team proposed applying synthetic biology methods to tuberculosis vaccines (ie, Project APRICOT [Antigen Presentation Using Crispr for TB] [58]).

**Table 2:** Co-Immune project descriptions.

Project name	Project status	Solution category	Summary description
HERA^a^: A Health Platform for Refugees [53]	Assessed^b^ Awarded Grand prize Best approach prize Best impact strategy prize	Software Knowledge transfer	A mobile health app designed for improving the monitoring of vaccination and perinatal health of Syrian refugees in Turkey; it provides recall of vaccines, storage of health data, health promotion (educational content), and financial incentives for immunization
Qualitative Analysis of Tweets on Vaccination [56]	Assessed Awarded Partner prize	Software Knowledge production	A web-based platform providing real-time visualization and analysis of tweets related to vaccination and vaccination hesitancy; data analysis included sentiment analysis and network analysis; an area of development was the development of predictive models of epidemic occurrence based on Twitter data
Commit to Get Vacc & to Promote Vaccination – HEROIC Santé [57]	Assessed Awarded Best implementation strategy prize	Intervention Knowledge transfer	A short questionnaire (7 minutes) using engagement approaches from the human and social sciences, such as “the importance of the source,” “voluntary consent,” or “fear and danger management,” to engage health care professionals and users, not only to be vaccinated against the flu, but also to promote flu vaccination
Project APRICOT^c^ [58]	Assessed Awarded Partner prize	Hardware	Development of a synthetic biology–based methodology that addresses the evasion mechanisms adopted by the mycobacterium tuberculosis and induces the acceleration of lysosomal biogenesis to improve antigen presentation
Vaccination Awareness Escape Game [54]	Assessed Not awarded	Intervention Knowledge transfer	An escape game to raise vaccination awareness among the general population
Harmonize Vaccination [59]	Assessed Not awarded	Software Knowledge production	A tool for parsing various formats of vaccination coverage data sets and for visualizing them on a common platform
Pass It On: A Game About Vaccine Hesitancy [60]	Assessed Not awarded	Software Knowledge transfer	A role-play video game aiming to improve the capacity of health professionals to respond to their patients’ hesitation to be vaccinated
Global Vaccination Risk Assessment [61]	Assessed Not awarded	Software Knowledge production	A tool to create an overview of risk factors of “not getting vaccinated,” by country, while looking at the more comprehensive picture; the methodology of this project is based on fuzzy logic, multi-criterion analysis, and the risk triangle
Immuno [62]	Not assessed^d^	Hardware Knowledge transfer	A board game providing access to the general public’s understanding of medical sciences related to immunization
Vaccine DataDump [63]	Not assessed	Software Knowledge production	A vaccination-related data repository and analysis tool for quick analysis of vaccine-related issues
Measuring Vaccination Hesitancy From Social Media [64]	Not assessed	Software Knowledge production	Data analysis of social media (ie, Twitter) to examine whether negative sentiment related to vaccination precedes declaration of symptoms and to study the relationship between vaccination hesitancy and epidemiological outbreaks
Mortality According to Access to Vaccines [65]	Not assessed	Software Knowledge production	Data analysis exploring the link between immunization coverage, mortality rate, and distance from health centers
The Health System Matrices [66]	Not assessed	Software Knowledge production	Exploratory analysis of the various parameters influencing vaccination coverage over time
Meta Immune – Data Exploration of Existing DB [67]	Not assessed	Software Knowledge production	A data lake on immunization data
Biloba^e^ [68]	Not assessed	Intervention	An intervention incentivizing people to increase vaccine uptake through vouchers, supporting the existing mobile app Biloba
Wakuchin Senshi [69]	Not assessed	Intervention Knowledge transfer	An interactive role-play board game to increase awareness about vaccination among the general population
Neutralizing Information About Vaccines [70]	Not assessed	Software Knowledge transfer	An algorithm for parsing web pages, identifying misinformation, and identifying trustworthy content to help users in their health decisions related to vaccines; this also aims to be used by search engines in their recommender systems
Go Viral! [71]	Not assessed	Intervention Knowledge transfer	A communication campaign on social media using gamification methods to illustrate contagion among users and, thereby, increase awareness of the importance of vaccines
Make Vaccines Affordable [72]	Not assessed	Software Knowledge transfer	A web-based portal with data related to population demand for care in order to negotiate prices of vaccines with suppliers
Identify Topics of Discussion in Vaccination Posts [73]	Not assessed	Software Knowledge production	Analysis of discussion in vaccination-related posts on Twitter and their evolution over time
Detect Vaccine Administration in Social Media Patient Data [74]	Not assessed	Software Knowledge production	A classifier able to detect vaccine administration in tweets related to vaccination
Detect Vaccine Hesitancy in Social Media Patient Data [75]	Not assessed	Software Knowledge production	A classifier able to detect vaccine hesitancy in tweets related to vaccination

^a^HERA: Health Recording App.

^b^These were projects that were assessed by experts at the end of the program. To be assessed by a pool of experts, the project team needed to provide detailed documentation of their project, provide a short video pitch, and deposit their data and code on the Just One Giant Lab (JOGL) platform.

^c^APRICOT: Antigen Presentation Using Crispr for TB.

^d^These were projects that were not assessed by experts at the end of the program because they did not provide sufficient documentation.

^e^The Biloba project, which was not part of Co-Immune, was used as a base to create the team’s own project, as the Biloba founder was a mentor during this event.

### Co-Immune Project Assessment

Out of 22 projects, 7 (32%) provided sufficient documentation on JOGL to be assessed by the pool of independent experts. In total, 27 reviews were performed, yielding scores ranging from 18 to 32.8 out of a possible total of 45 across the different dimensions that were assessed (ie, approach, implementation strategy, and impact). The average score was 25.1 (SD 6.4).

HERA: A Health Platform for Refugees [53] was awarded with prizes, based on a total score of 15, for best approach (mean score 11.4, SD 2) and impact (mean score 14.6, SD 3.2). Commit to Get Vacc & to Promote Vaccination – HEROIC Santé [57] was awarded the best implementation strategy prize (mean score 10.33, SD 2.5).

**Figure 8:**
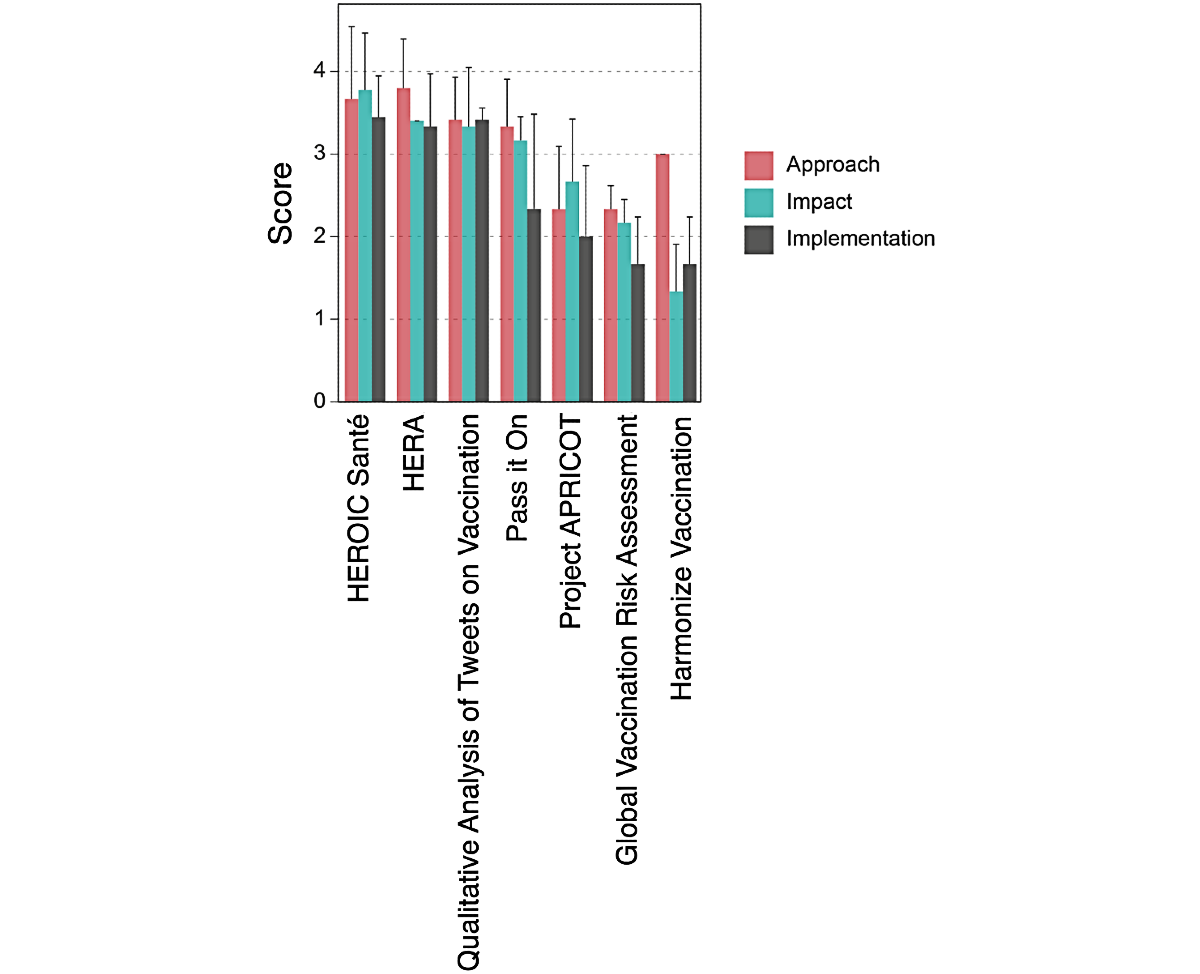
Bar plot of review scores per category for all reviewed projects. Bars show average values for all questions related to each category, and error bars represent SDs. Projects are shown by decreasing global score. APRICOT: Antigen Presentation Using Crispr for TB; HERA: Health Recording App.

The projects were more successful, globally, in terms of approach, with a mean score of 9.37 (SD 1.79) out of 15 points. Out of 7 projects that were assessed, 4 (57%; Figure 8) had a score higher than 4 out of 5 for clarity, relevance, and alignment of their problem statement with the program objectives. For 6 projects (86%), the fit between the methods and the projects’ objectives was scored highly by reviewers, with a score of at least 3 out of 5.

The implementation strategy score of projects was low, overall, given the early stage of the projects at the time of review. As such, only projects that existed prior to the program—HERA [52] and HEROIC Santé [57]—got a score of at least 3 out of 5.

For winners in each category, JOGL awarded them physical space for showcasing their project during the 2020 ChangeNOW forum at the Grand Palais in Paris as well as tickets for the Maddy Keynote, a major innovation event in Paris. Two partners—Excelya and the Wild Code School—also provided awards to the projects of their choice. Additionally, the Qualitative Analysis of Tweets on Vaccination [56] project was chosen to be the focus of a hackathon by the Wild Code School, and Project APRICOT [58] was offered technical support for data science and legal and regulatory affairs by Excelya.

## Discussion

### Principal Findings

The Co-Immune program was designed to foster the creation and development of citizen science and open innovation projects addressing the contemporary challenges of vaccination in France and around the globe by reaching four specific objectives: (1) to foster collaborative, open, and transdisciplinary dynamics; (2) to promote the emergence of accessible knowledge and innovative solutions; (3) to support participants in the elaboration and development of their projects; and (4) to disseminate the outputs and results in an open science framework. Below, we discuss to what extent Co-Immune reached these objectives and highlight the challenges and facilitators in implementing such a program.

First, the program succeeded in creating a collaborative and transdisciplinary environment through its three core features: the JOGL platform, the organization of events, and the contest approach. This led to forming partnerships with 13 different organizations and recruiting over 230 participants, who displayed 492 unique skills and were engaged in creating 22 projects. The use of on-site hackathons was beneficial in gathering nonacademic participants from various backgrounds. Our data show that in-person events and local outreach played a significant role in growing the community around Co-Immune. These offline events recruited 45% of the total community. Local enrollment was further strengthened by local partnerships, such as higher education organizations. However, the localization of our on-site events in Paris did not allow for the participation of people living in other parts of France or the rest of the world. Additionally, our online communication restricted the access of the online events to our realm of influence and to people with an internet connection. More inclusive participation geared toward people with diverse socioeconomic statuses and geographic situations is desirable in the future to give them agency over solving the problems that affect them. The development of new communities is usually a slow process in the absence of exogenous shocks, such as the surge in collaborative communities created by the COVID-19 pandemic [76]. Tapping into existing projects and networks for events has proven to be fruitful in our case, allowing for a steady growth of the Co-Immune community up until the end of the program. However, we did not observe further growth of the community after the end of the program. This highlights that in order to build a sustainable community using open innovation to tackle global health challenges, one needs to facilitate the entry and exit of members, provide resources to support the current ones, focus on building on existing communities and projects, design inclusive environments for collaboration, and empower members to run their own activities.

Second, two design elements of the program converged to promote the emergence of knowledge and solutions to address aspects of access to vaccines and vaccination hesitancy: (1) the identification of challenges by experts in the field and (2) the alignment of the program strategy with national and international policies by frequent consultation with public health bodies and mobilization of members of public institutions in the CESI. Yet, greater representation of people affected by poor access to vaccines and people who are hesitant would be desirable to strengthen the alignment between the solutions developed and the most pressing needs at the local level.

Recently, online events have been used widely during the COVID-19 pandemic [76-78], supporting our initial assumption that forming and animating a distributed online community for public health programs is a relevant approach.

Third, the use of the JOGL platform, the mentorship during events, the assessment and feedback from experts, and the connection with a wide range of partners supported participants in the elaboration of their project in an efficient way. The use of the JOGL platform enabled projects to gain visibility, list their needs to create interfaces for collaboration, and share open data sets, code, and tools. Indeed, online platforms can offer projects that started at hackathons a pathway to pursue their development, potentially alleviating one of the main drawbacks of such short temporal interventions [43]. In this case, it also enabled the program coordinators to connect participants with project leaders based on a match between needs and skills. Yet, this approach was time-consuming, and scaling up our efforts proved to be challenging. The automation of such matchmaking tasks through a recommender system would help to minimize these efforts and increase the impact of projects through accelerated development [79]. In addition, mentoring is a known strategy that is used by open, online communities [80,81] and was leveraged by the Co-Immune program. Given the diversity of backgrounds and level of expertise across the participants, it was necessary to engage a similar diversity among the mentors. In our context, the highly rated projects that eventually received awards did not originate or participate in hackathons, but rather benefited from Co-Immune as a platform for further growth. Several of these projects already existed before the start of Co-Immune and had a higher maturity level than the projects created during the short span of the program. In addition, these projects were launched and run by people outside the larger Paris region. Thus, we stress the potential of online platforms and open innovation to build on existing projects and to replicate, adapt, and scale their activities in other contexts. Additional support consisted of promoting visibility on social media by the organization team as well as opportunities for networking during events. Although no financial compensation was provided as part of this program, partners, through their own experts and co-organizing events, engaged in close relationships with JOGL and the individual projects. This was favorable for sustaining collaborations and projects after the end of the program. In the future, the sustainability of the newly created project efforts could potentially be improved by using incentives, such as microgrants or fellowship programs, for continuing projects in the postprogram period [79]. While the short time frame and limited resources allocated to the program did not allow us to implement a strong monitoring and evaluation strategy, future implementations should ensure that they conduct a minimum of pre- and postprogram data collection for assessing the full impact of the program.

Finally, the open science environment of this program was not only an asset for disseminating the outputs and results of the projects developed, but it also enabled them to replicate initiatives and, thereby, accelerate the resolution of the global health challenges they address. An example of this was given by the team from the project HERA: A Health Platform for Refugees [52], who opened its code, enabling any individual to replicate it. However, the lack of a thorough evaluation strategy prevents us from reaching a more definitive conclusion on the effective replication of projects carried out in Co-Immune.

Co-Immune showcases that short, focused programs can be efficient at mobilizing diverse communities in a rapid manner and harvesting ideas from various domains to address global health challenges. Yet, more case studies and evaluation work on similar programs are necessary to assess the full relevance of their design and the impact of the projects that are developed within them.

### Conclusions

Co-Immune highlights how open innovation approaches and online platforms can help to gather and coordinate noninstitutional communities in a rapid, distributed, and global way toward solving SDG-related issues. The Co-Immune program gathered participants and partners from various backgrounds in a newly formed community to facilitate the creation of new projects as well as the continuation of existing projects to address the issues of vaccination hesitancy and access. In an open framework, the projects made their data, code, and solutions publicly available.

Through hackathons and other contest approaches, such initiatives can lead to the production and transfer of knowledge, creating novel solutions in the public health sector. The example of Co-Immune contributes to paving the way for organizations and individuals to collaboratively tackle future global challenges.

## Appendix

Multimedia Appendix 1 Co-Immune partners and supplementary method for comparing Just One Giant Lab (JOGL) with other platforms.

Multimedia Appendix 2 Co-Immune project assessment grid.

## Abbreviations

APRICOT: Antigen Presentation Using Crispr for TB
CESI: Committee for Ethics, Science and Impact
CRI: Center for Research and Interdisciplinarity
HERA: Health Recording App
JOGL: Just One Giant Lab
SDG: Sustainable Development Goal
WHO: World Health Organization
