# Supplementary material for: Implementing the Co-Immune Open Innovation Program to Address Vaccination Hesitancy and Access to Vaccines: Retrospective Study
Source: J Particip Med. 2022 Jan 21;14(1):e32125. doi: 10.2196/32125 (PMC8817221; doi:10.2196/32125)
Supplement: Multimedia Appendix 1 [file jopm_v14i1e32125_app1.pdf]

# Multimedia Appendix 1

## Table S1. Co-Immune partners.

| Partners | Status | Activity |
|---|---|---|
| Sanofi | Private | Healthcare Industry |
| S3 Odéon | Non-profit | Communication And Event |
| Epitech | Private | Education |
| CorrelAid | Non-profit | Innovation And Technology |
| SUP biotech | Private | Education |
| Excelya | Private | Research |
| Wild Code School | Private | Education |
| Girls in Tech | Non-profit | Innovation And Technology |
| Kap Code | Private | Innovation And Technology |
| TUBA | Non-profit | Incubator |
| Data for good | Non-profit | Incubator |
| Maddy Keynote | Private | Communication And Event |
| Change Now | Private | Communication And Event |

# Supplementary method: Comparing JOGL with other platforms

## *Selection of comparative platforms*

In order to situate the action and suitability of Just One Giant Lab (JOGL) as a host for Co-Immune, we selected popular social networks, science publishing and collaboration websites, and citizen science and project creation websites as elaborated on below.

*Social networks*: We selected platforms for which the primary focus is the connection of people and thoughts, without focus on projects or third person entities- they are dedicated to the self, and fulfil social needs, from chatting to self-promotion and networking [1]: LinkedIn, Instagram, Quora, Facebook, Twitter, Discord, Reddit, and Mind.

*Science publishing and collaboration sites*: These platforms are not focused on the self, but instead on third person goals such as manuscript edition and curation, project creation or grant acquisition. We selected: Nature, Open Peer Review MNI, OSF collaboration, BioRxiv, PubPub, EasyChair, Je-S system, ResearchGate, Academia, Mendeley, and mySociety (RSB) [2-4].

*Citizen science and project creation sites*: These tools enable a commons-based peer production, using open science, crowdsourced data collection, collaboration or challenge-based approaches: Kaggle, Hackaday, Experiment, Open Humans, Zooniverse, Instructables, Wikipedia, and FLOSS Manuals [5,6].

## *Social network feature comparisons method*

We evaluated the presence (1) or absence (0) of 42 features across these platforms that were manually selected for their relevance with the organisation of the Co-Immune programme. Features encompass communication, collaboration, and participant behavior. Platform clustering was performed using correlation distance and average linkage method. In order to gain insights on the proximity between platforms and take into account correlation between features, we performed a principal component analysis (PCA) and projected platform feature vectors on the top two eigenvectors (Figure S1). This allowed us to visualise the 3 types of platforms (colors). The top eigenvector, concentrating most of the variance across features, segregates social networks from scientific and project-based platforms. The second component in turn separates publication from peer-production platforms. This allows us to situate JOGL in a central position with respect to the first component (mixing science and social network), while clustering it with peer-production platforms.

Figure S1. JOGL’s position as a social network for open science.

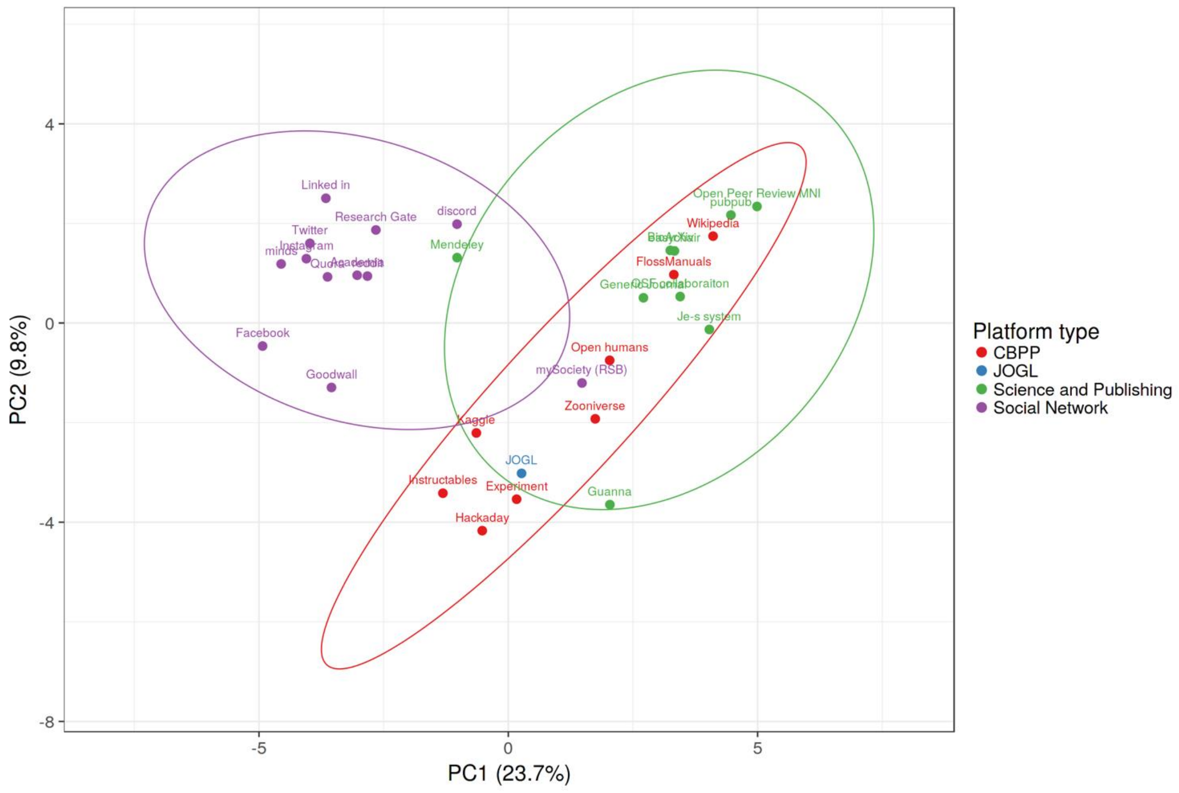


Various online platforms represented in the first 2 components of a PCA based on their features from Figure 1. We use the pcaMethods R package with default parameters to calculate principal components. Catchment of groupings represent a 95% certainty interval of a platform landing within the platform type through feature space. N = 29 platforms, each with 42 true/false data points. CBPP- Citizen-based peer production network.

## *References*


1. Bolar KP. Motives behind the use of social networking sites: An empirical study. IUP J Manage Res 2009 Jan; :1-10.
2. Ross-Hellauer T, Deppe A, Schmidt B. Survey on open peer review: Attitudes and experience amongst editors, authors and reviewers. PLoS One 2017; 12(12):e0189311.
3. Van Noorden R. Online collaboration: Scientists and the social network. Nature 2014 Aug 14; 512(7513):126-129.
4. Joint Electronic Submissions (Je-S). Je-S Handbook [accessed 2020-10-09]. https://je-s.rcuk.ac.uk/Handbook/Index.htm.
5. Cohn JP. Citizen science: Can volunteers do real research?. Bioscience 2008 Mar; 58(3):192-197
6. Benkler Y, Nissenbaum H. Commons-based peer production and virtue. J Polit Philos 2006; 14(4):394-419.
