# Supplementary material for: Implementing the Co-Immune Open Innovation Program to Address Vaccination Hesitancy and Access to Vaccines: Retrospective Study
Source: J Particip Med. 2022 Jan 21;14(1):e32125. doi: 10.2196/32125 (PMC8817221; doi:10.2196/32125)
Supplement: Multimedia Appendix 2 [file jopm_v14i1e32125_app2.pdf]

Co-Immune | Project Assessment

Congratulations for being part of our reviewers! You are going to play a very important role in the future of projects that could help humanity solve one of its great challenges, better health for all. Thank you!

Co-Immune is an open and collaborative research and innovation program carried out by the NGO Just One Giant Lab, which aims to contribute to improving vaccination coverage in France and across the globe. (More on app.jogl.io/program/coimmune)

Scores you attribute will be used by the honorary and independent Committee for Ethics, Science and Impact (CESI) of the Co-Immune program to attribute prizes on the 18th of December in Paris during the Co-Immune ceremony. Register for the event here http://bit.ly/co-immune_ceremony !

More information on the evaluation framework and prizes here: http://bit.ly/assessment-framework

WHEN?
This form is to be completed by the 16th or the 17th of December.

WHY?
This form will allow you to evaluate the projects submitted to the Co-Immune program, The scores and comments given will be used to assess the approach, implementation, and impact of each project.

WHAT?
We kindly request you to provide independent, fair, unbiased and constructive feedback. No conflict of interest with the Co-Immune financing partner shall be accepted.

HOW?
Assessment is made anonymously. JOGL will not share your name or contact information to any participant.
The comments given here will be made public on the platform. However, the given scores will be hidden to the public. Your identity will remain anonymous unless you purposely waive your anonymity.

QUESTIONS ?
If you have any questions, please, feel free to contact JOGL at hello@jogl.io

READY? GO!

* Required

1. Email *

2. Your first name and last name *

This information is only used by JOGL for admin purpose and will not be made public. This evaluation is anonymous.

3. Please provide information regarding your current or previous links of interest with Sanofi, the main financial partner of the Co-Immune program. *

Mark only one oval.

- I certify I have no link of interest with Sanofi
- I have links of interest with Sanofi. Therefore, I regrettably cannot take part in the project assessment

4. Link of the project page being reviewed *

You can find the list of links and names of projects to review here: http://bit.ly/active-projects-links

Project Approach

In this section, you will assess the approach of the project. Please rank from 1 to 5 each criteria below. You can provide additional comments in the appropriate field.

5. The clarity and relevance of the problem that the project is attempting to solve and alignment with the Co-Immune program's scope [Score] *

1. Unclear and/or irrelevant | 3. Clear but relevant points are missing | 5. Very clear and relevant

Mark only one oval.

1	2	3	4	5

6. The clarity and relevance of the problem that the project is attempting to solve and it's alignment with the Co-Immune program's scope [Comment, optional]

Please, comment and explain the score you have given. You can also give constructive feedback to help the project improve on this criteria.

7. Fit between the project's approach/methodology and the problem they have stated to resolve [Score] *

1. Inappropriate | 3. Appropriate | 5.Very appropriate

Mark only one oval.

1	2	3	4	5
⬭	⬭	⬭	⬭	⬭

8. Fit between the project's approach/methodology and the problem they have stated to resolve [Comment, optional]

Please, comment and explain the score you have given. You can also give constructive feedback to help the project improve on this criteria.

9. Introduction of ground-breaking objectives, novel concepts or approaches by the project [Score] *

1. Not innovative | 3. Innovative | 5. Disruptive

Mark only one oval.

1	2	3	4	5
⬭	⬭	⬭	⬭	⬭

10. Introduction of ground-breaking objectives, novel concepts and approaches [Comment, optional]

Please, comment and explain the score you have given. You can also give constructive feedback to help the project improve on this criteria.

Implementation

In this section, you will assess the implementation strategy of the project. Please rank from 1 to 5 each criteria below. You can provide additional comments in the appropriate field

11. The project's state of progress [Score] *

1. No results yet | 3. Some results but with a promising development plan | 5. Working proof of concept

Mark only one oval.

1	2	3	4	5
⬭	⬭	⬭	⬭	⬭

12. The project's state of progress [Comment, optional]

Please, comment and explain the score you have given. You can also give constructive feedback to help the project improve on this criteria.

13. Clarity and relevance of the project timeline and it's needs for future (major tasks, milestones) [Score] *

1. Unclear and irrelevant | 3. Clear but relevant points are missing | 5. Very clear and relevant

Mark only one oval.

1	2	3	4	5
⬭	⬭	⬭	⬭	⬭

14. Clarity and relevance of the project timeline and it's needs for future (major tasks, milestones) [Comment, optional]

Please, comment and explain the score you have given. You can also give constructive feedback to help the project improve on this criteria.

15. The project's ability to actively engage and align itself with all the relevant groups and possible stakeholders [Score] *

1. Unaware | 3. Aware but unclear integration | 5. Actively engaged

Mark only one oval.

1	2	3	4	5
◯	◯	◯	◯	◯

16. Ability to actively engage and align itself with all the relevant groups and possible stakeholders [Comment, optional]

Please, comment and explain the score you have given. You can also give constructive feedback to help the project improve on this criteria.

Impact

In this section, you will assess the impact of the project. Please rank from 1 to 5 each criteria below. You can provide additional comments in the appropriate field.

17. Clarity and relevance of the criteria used to measure impact [Score] *

1. Unclear and irrelevant | 3. Clear but not entirely appropriate | 5. Very clear and relevant

Mark only one oval.

1	2	3	4	5
◯	◯	◯	◯	◯

18. Clarity and relevance of the criteria used to measure impact [Comment, optional]

Please, comment and explain the score you have given. You can also give constructive feedback to help the project improve on this criteria.

19. To what extent does the project takes into account its ecosystem (ecological, environmental, ethical and social considerations) [Score] *

1. Unaware | 3. Aware | 5. Very aware

Mark only one oval.

1	2	3	4	5
⬭	⬭	⬭	⬭	⬭

20. To what extent does the project takes into account its ecosystem (ecological, environmental, ethical and social considerations) [Comment, optional]

Please, comment and explain the score you have given. You can also give constructive feedback to help the project improve on this criteria.

21. The sustainability and scalability of the project in the long term [Score] *

1. No sustainability model and no scaling potential | 3. Sustainable and scalable but still unstructured plan | 5. Already applying a sustainable plan & good scalability

Mark only one oval.

1	2	3	4	5
⬭	⬭	⬭	⬭	⬭

22. The sustainability and scalability of the project in the long term [Comment, optional]

Please, comment and explain the score you have given. You can also give constructive feedback to help the project improve on this criteria.

____

____

____

____

____

23. The project's dissemination strategy (quality of documentation for goals, results, methods and needs; open access model, outreach) [Score] *

1. Irreproducible and poorly documented 3. Good documentation but with few weaknesses 5. Easily reproducible & very well communicated

Mark only one oval.

1	2	3	4	5
⬭	⬭	⬭	⬭	⬭

24. The projects dissemination strategy (quality of documentation for goals, results, methods and needs; open access model, outreach) [Comment, optional]

Additional comments and reviewer self-evaluation

You may provide below additional comments addressed to the team leaders of the project, and private comments to the JOGL team.

Please, also assess your expertise in the fields relevant to evaluate the project.

25. [To the team leaders] Other general constructive comments about the project *

26. [To JOGL] Private comment - anything you would like to inform the JOGL team of, privately.

27. What is your expertise that is relevant to evaluate this project? *

Please provide justification of your expertise if possible.

28. Would you like us to include you in our "Experts community" and provide similar reviews to projects when you have time in the future? *

Mark only one oval.

- Yes
- No
- Maybe

Thank you very much for your feedback!

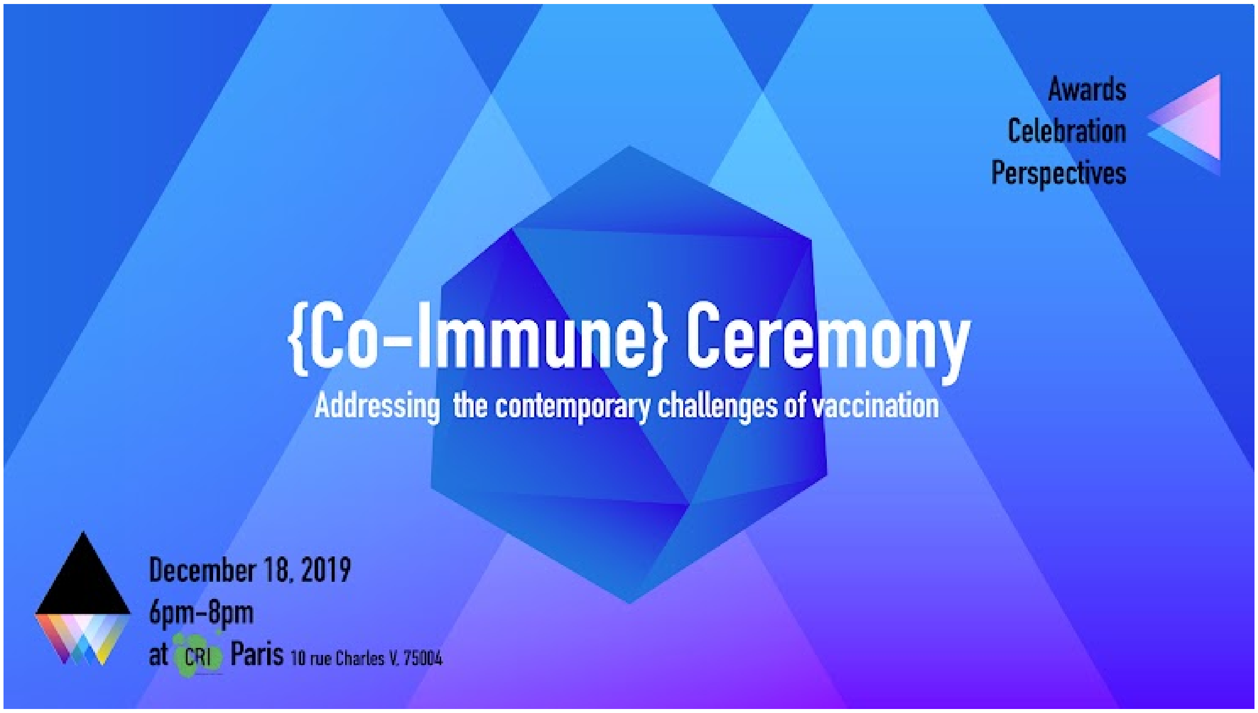

Just One Giant Lab
learning & solving together

This content is neither created nor endorsed by Google.

Google Forms
